# Artificial Intelligence-Driven Sensing Framework with Multimodal Sensor Importance Learning for Smart Energy Systems

**DOI:** 10.3390/s26092791

**Published:** 2026-04-30

**Authors:** Shujin Zhang, Zhuochen Liu, Kai Sun, Yueyang Wang, Xiaohan Hu, Zhonghao Zhang, Yan Zhan

**Affiliations:** 1 National School of Development, Peking University, Beijing 100871, China; 2 China Agricultural University, Beijing 100083, China; 3 Beihang University, Beijing 100191, China; 4 Artificial Intelligence Research Institute, Tsinghua University, Beijing 100084, China

**Keywords:** artificial intelligence-driven sensing, multimodal data fusion, multimodal sensors, smart energy systems, spatiotemporal time-series modeling

## Abstract

Against the background of accelerated green energy development and the deep integration of intelligent sensing technologies, wind power forecasting is evolving toward a multimodal sensor collaborative perception paradigm within nonlinear multi-source integrated energy systems. To address the limitations of conventional methods, including the lack of dynamic importance modeling and constrained stability under complex wind conditions, a forecasting framework based on multimodal sensor importance perception is proposed. This study emphasizes the framework’s role in decoding the complex nonlinear dependencies between atmospheric drivers and turbine responses. Through a multimodal feature encoding architecture, unified temporal representations of meteorological environments and turbine operational states are established. A sensor-importance-aware attention mechanism and a cross-modal relational modeling strategy are introduced to adaptively allocate contributions under varying contexts. Furthermore, prediction compensation and uncertainty characterization modules are integrated to enhance robustness. Systematic experiments on real-world multi-source data validate the method. Overall performance comparisons demonstrate that MAE, RMSE, and MAPE reach 30.48, 42.37, and 9.16 percent, respectively, with the coefficient of determination $R^{2}$ achieving 0.957, significantly outperforming the Transformer baseline. In multi-horizon tasks, the model exhibits superior error accumulation suppression, with twelve-step forecasting errors remaining at 41.27 and 56.48. These findings reveal that the framework captures the context-dependent nonlinear mapping of energy systems, providing effective technical support for green energy dispatch and intelligent sensing applications.

## 1. Introduction

The global energy system is accelerating toward a low-carbon electricity structure, and wind energy has become one of the most scalable energy options in this transition [1,2]. However, wind power output is inherently characterized by randomness, intermittency, and non-stationarity, which renders unit commitment, reserve allocation, and real-time balancing control in modern power systems more complex [3,4]. This challenge is particularly prominent in short-term and ultra-short-term forecasting, because dispatch decisions often must be completed before uncertainty can be smoothed over longer temporal horizons [5,6]. Therefore, wind power forecasting is not merely a statistical problem, but rather a critical enabling technology for ensuring secure grid operation, promoting renewable energy accommodation, and realizing economic dispatch [7]. Existing studies have repeatedly demonstrated that forecasting errors increase balancing costs, induce more conservative wind curtailment decisions, and weaken operational confidence in renewable energy dispatch [8]. The rapid development of the Industrial Internet of Things (IIoT) has also profoundly transformed the observation and modeling paradigm of wind farms [9,10]. Modern wind farms continuously generate high-density heterogeneous data streams from supervisory control and data acquisition (SCADA) systems [11], numerical weather prediction (NWP) products [12], and meteorological towers or station-level weather stations [13]. These modalities jointly characterize turbine operating conditions [14], environmental meteorological states, large-scale atmospheric evolution, and local wake-related responses [15]. Compared with the use of historical power sequences alone, multimodal sensing is expected to more comprehensively represent the causal chain from atmospheric drivers to turbine output [16]. However, richer sensing does not automatically translate into higher forecasting accuracy, because multimodal data simultaneously introduce substantial heterogeneity in sampling frequency, noise distribution, spatial coverage, and semantic attributes [17,18].

Despite the rapid development of data-driven wind power forecasting methods, two particularly prominent limitations remain in multimodal scenarios [19,20]. First, static fusion is still widely adopted. Many studies directly concatenate heterogeneous sensor features after resampling, or feed them into a shared encoder with fixed channel interactions [21]. Such designs implicitly assume that the contributions of each modality to the forecasting target remain approximately constant across different time periods, weather scenarios, and operating states [15,22,23]. However, in real wind power operations, sensor effectiveness is highly context-dependent. For example, under stable operating conditions, historical power and turbine status variables may dominate; by contrast, before ramp events or abrupt weather changes, pressure gradients, wind direction deviations, or NWP-derived frontal information may become more discriminative [24]. As a result, fixed fusion rules often underestimate critical modalities while amplifying redundant or noisy ones [25]. Second, model interpretability remains insufficient [21]. Although deep recurrent networks, convolutional networks, and Transformer-based models often improve benchmark accuracy, their internal decision logic generally remains a black box for dispatchers and operation personnel [26,27]. Post-hoc explanation methods may provide certain clues, yet recent studies in the wind power domain have shown that model-agnostic explanation methods may not always remain stable or trustworthy in safety-sensitive scenarios [28,29]. Consequently, a clear trust gap still exists between high offline accuracy and actual operational adoption.

To address these issues, an interpretable wind power forecasting framework based on an attention mechanism and multimodal sensor-importance awareness is proposed in this study. The core idea is to explicitly model the time-varying relevance of the *m*-th sensing modality at time step *t* through a dynamic weight $\alpha_{t}^{m}$. Rather than treating SCADA, NWP, and meteorological observations as equally important inputs, the proposed framework adaptively learns which modality should receive greater weight according to the current wind condition and temporal context. This design serves two goals. First, complementary information is exploited only when it is genuinely valuable, thereby reducing the inefficiency of static concatenation and fixed fusion while improving forecasting performance. Second, interpretability is embedded directly into the forecasting pathway, allowing the learned modality weights to serve as endogenous sensor-importance signals rather than externally appended post-hoc explanations. In this way, the attention mechanism functions not only as a feature interaction tool, but also as a structured interface between data-driven learning and operational cognition. The main contributions of this study are summarized as follows:

1. A dynamic multimodal sensor-importance-aware forecasting paradigm is developed, in which heterogeneous observations are fused through context-dependent modality weighting rather than static concatenation, thereby enabling the model to dynamically adjust its information acquisition strategy as wind conditions evolve.
2. By explicitly associating the learned weights $\alpha_{t}^{m}$ with the forecasting process, an endogenous interpretability mechanism is introduced, allowing the sensing modalities that dominate the prediction to be traced during inference and linked to wind farm operating states.
3. Systematic validation is conducted on a real-world multi-source wind farm sensing dataset, and the results demonstrate that dynamic modality awareness not only improves forecasting accuracy and robustness, but also enhances the practical application value of model outputs in grid-side dispatch and green energy management.

## 2. Related Work

### 2.1. Wind Power Forecasting Methods

Wind power forecasting has evolved from classical stochastic models to modern deep learning architectures capable of modeling complex temporal and spatial dependencies [30]. This progression is largely driven by the increasing nonlinearity of high-penetration wind power data and the growing availability of high-frequency operational and meteorological observations [31]. Early approaches, including autoregressive integrated moving average (ARIMA) [32], generalized autoregressive conditional heteroscedasticity (GARCH) [33], and related linear models, are computationally efficient and interpretable, but their performance degrades under strong nonlinearity, multi-scale variability, and dependence on exogenous factors [34]. To address these limitations, machine learning methods such as support vector regression [21], Gaussian process regression, and tree-based models have been widely adopted [35]. These approaches improve nonlinear mapping between meteorological inputs and power output, particularly when combined with domain-informed features [36]. For example, Gaussian processes offer advantages in uncertainty quantification under limited data scenarios, while tree-based and kernel methods remain competitive for moderate sample sizes [36]. However, their reliance on manual feature engineering and limited capacity to capture long-term dependencies and cross-variable interactions constrain their scalability to complex, multi-source forecasting tasks [37]. Deep learning further alleviates the need for manual feature design by enabling hierarchical representation learning from raw sequences [9]. Architectures such as convolutional neural networks [14], recurrent neural networks [25], long short-term memory networks [21], and gated recurrent units have demonstrated superior performance in modeling nonlinear dynamics and temporal dependencies in wind power forecasting [38]. More recently, Transformer-based architectures have reshaped the field by improving long-sequence modeling efficiency and flexibility [38]. Informer introduces ProbSparse self-attention to reduce computational complexity [28], while Autoformer leverages decomposition and autocorrelation mechanisms to capture trend–seasonality interactions [26]. Despite these advances, a critical gap remains between predictive accuracy and operational interpretability [27]. Existing models, including attention-based architectures, primarily focus on temporal steps [39], latent features, or spatial nodes, but rarely provide explicit insight into the relative contributions of heterogeneous sensing modalities such as SCADA [40], numerical weather prediction, and meteorological observations [41,42]. This limitation suggests that future research should move beyond increasing model complexity toward multimodal frameworks capable of dynamically quantifying modality-level importance [24]. Addressing this gap constitutes a key motivation for the proposed approach [43].

### 2.2. Multimodal Sensor Data Fusion in Wind Power Forecasting

With the continued deployment of SCADA systems, meteorological towers, and external weather products [14], data fusion has become a central challenge in wind power forecasting research [15]. Existing approaches can be broadly categorized into three paradigms: early fusion, late fusion, and intermediate fusion [12]. Among these, early fusion has been widely adopted due to its simplicity and its ability to preserve raw covariate information [38]. For example, some studies that simultaneously utilize weather simulation outputs, weather regime information, and SCADA observations generally perform feature-level concatenation first and then input the fused sequence into a shared backbone network [12,15]. Early fusion may be effective when multimodal synchrony is relatively good, but it may also obscure the semantic boundaries among different sensing channels [7]. Late fusion follows another line of reasoning, in which independent predictors are established for different data sources, and the outputs of these branches are combined at the decision level [36]. Intermediate fusion attempts to address the above shortcomings by enabling modality interactions within the latent representation space rather than performing one-time combination only at the input or output layer [40,41]. Attention-based collaborative models [24], graph-enhanced architectures [43], and hierarchical fusion frameworks have increasingly adopted this strategy in recent years [42,44]. Another persistent challenge arises from temporal alignment [15]. SCADA data are typically sampled at second-level or minute-level intervals, meteorological towers may follow different sampling rhythms [12], and NWP products are usually updated at coarser temporal resolutions [44]. In practical applications, these asynchronous data streams are often aligned through interpolation, aggregation, or fixed sliding windows [13]. Although such operations are convenient for engineering implementation [40], they may distort cross-modal relationships by smoothing informative high-frequency signals or introducing redundant low-frequency noise [39]. Unfortunately, existing fusion studies have rarely treated such differences in temporal reliability as explicit modeling objects, and even more rarely transformed them into learnable importance allocation mechanisms [26]. Therefore, the true challenge lies not only in how to fuse multimodal data, but also in how to determine which modality should be trusted more under the current spatiotemporal and physical context. Overall, multimodal fusion in wind power forecasting has evolved from simple concatenation to more sophisticated latent interaction paradigms, yet two key issues remain insufficiently resolved. First, most methods still lack explicit dynamic modality weight allocation mechanisms. Second, temporal alignment is still handled mainly through preprocessing rather than adaptive inference. These limitations indicate that a more appropriate framework should be capable of learning modality importance online and exposing this process as an interpretable basis for decision-making. This consideration directly motivates the modality-aware attention strategy adopted in the present study.

### 2.3. Attention Mechanisms and Interpretable Deep Forecasting Models

In intelligent energy forecasting, interpretability has become a critical requirement, particularly when model outputs are directly used for dispatch support and other risk-sensitive operational decisions [45]. Existing studies can be broadly divided into two categories: post-hoc explanation and self-interpretability [46]. Post-hoc explanation methods attribute or approximately explain the behavior of an already learned predictor after model training has been completed [29]. Among them, the most widely used approaches include Local Interpretable Model-Agnostic Explanations (LIME) and SHapley Additive exPlanations (SHAP) [47]. LIME approximates the local decision boundary through a simpler surrogate model, whereas SHAP assigns feature contributions to individual predictions based on a game-theoretic framework [45]. Because such methods can be attached to black-box models without modifying the original model architecture, they have been widely applied in energy forecasting and power system analysis tasks [47].

However, post-hoc explanation methods also suffer from several important limitations [43]. First, they explain a trained predictor from outside the model rather than making the model itself transparent. Second, their explanations may be influenced by perturbation strategies, background distributions, or surrogate approximation schemes, thereby reducing consistency across different operating conditions [46]. Transformer studies incorporating domain knowledge have further suggested that structured inductive bias may enhance the physical meaning of learned attention [24]. Beyond attention, gating-based strategies have also gradually emerged as a noteworthy direction. A recent study on SCADA data learned sparse predictors through gated lag and feature selection mechanisms [48]. Similarly, an interpretable day-ahead wind power forecasting framework based on SHAP and mixture of experts (MoE) has shown that expert selection weights can also provide meaningful explanatory layers under different meteorological scenarios [49].

Despite these advances, a clear differentiation must be made between existing weighting approaches and the proposed framework. As summarized in Table 1, most existing attention-based models primarily focus on temporal dependencies or variable-level importance after features have already been mixed [27,41]. In contrast, our approach introduces a modality-level self-interpretability mechanism that functions before and during the fusion stage. While general temporal attention addresses which time steps are critical [28], and feature selection ranks individual variables [48], the proposed sensor-importance-aware attention explicitly answers which sensing modality should be trusted under specific wind conditions [1]. Furthermore, unlike static modality weighting or fixed fusion rules, our method embeds a cross-modal relational modeling strategy to adaptively allocate information contributions. This conceptual shift from token-level focus to modality-level integrity check defines the unique positioning of this work in the current literature.

If a model can learn dynamic modality weights, then it may simultaneously answer two critical operational questions: how heterogeneous sensing sources should be fused to achieve high-accuracy prediction, and which class of sensing source is most critical to the final prediction under the current context [28]. Such a mechanism differs from post-hoc explanation because the weight allocation process is embedded within the forecasting pathway itself [26]; it also differs from general temporal attention because its explanatory target is the sensor modality itself [1]. It is precisely this conceptual distinction that defines the innovative positioning of the proposed method and provides the direct theoretical foundation for the sensor-importance-aware attention framework developed in this study.

## 3. Materials and Methods

### 3.1. Data Collection

The dataset utilized in this study was collected from the operational monitoring system of a large grid-connected wind farm located in northern China. Data acquisition was conducted through the coordinated operation of the supervisory control and data acquisition (SCADA) system deployed in the wind farm and the meteorological observation tower, covering two major categories of multimodal sensor data, namely meteorological environmental information and turbine operational status data, as shown in Table 2. Meteorological sensors were primarily installed on the central meteorological tower of the wind farm as well as on nacelle-mounted measuring devices of selected turbines, including key atmospheric variables such as wind speed, wind direction, ambient temperature, relative humidity, and air pressure. Wind speed and wind direction were measured using ultrasonic anemometers capable of three-dimensional vector acquisition, enabling high-precision sensing without mechanical inertia delay. Temperature and humidity observations were obtained through integrated digital thermo-hygrometers and were shielded by radiation-proof louver boxes to mitigate environmental interference. Atmospheric pressure was continuously recorded by high-stability barometers. All meteorological observations were automatically transmitted to the wind farm data center server at a sampling interval of 10min and were temporally aligned with turbine operational data through unified timestamps.

Turbine operational data were likewise recorded in real time by the SCADA system, including key parameters such as active power output, rotor speed, generator speed, nacelle temperature, gearbox temperature, and yaw angle. These operational sensors were embedded within the turbine control subsystems and transmitted to the central monitoring platform via industrial ethernet communication networks. Power output measurements were directly obtained from electric energy metering modules with high metrological precision, accurately reflecting turbine generation capacity. Rotational speed variables were collected through encoders and rotational speed sensors, enabling the characterization of mechanical response dynamics of turbine systems. Temperature measurements were realized through multi-point thermocouple deployments to continuously monitor thermal states of critical mechanical components. Operational data and meteorological observations were synchronously collected under a unified temporal reference, ensuring temporal consistency across multimodal sensor streams.

A continuous 24-month historical dataset was selected as the raw data foundation for this study, covering the period from January 2022 to December 2023. The dataset comprehensively reflects wind power variation characteristics under diverse seasonal patterns and wind conditions. To address concerns regarding generalizability, it is important to note that this 24-month span encompasses a vast range of meteorological extremes, including seasonal monsoon transitions, abrupt temperature fluctuations, and varying atmospheric stability classes typical of northern China. While the data originate from a single wind farm, the high-frequency sampling and long-term coverage provide a statistically representative environment for modeling complex nonlinear dynamics. After undergoing data preprocessing procedures including noise filtering, outlier removal, and missing value imputation, a structured multimodal wind power forecasting dataset was constructed. Furthermore, a 5-fold cross-validation strategy was implemented during model development to ensure that the findings are not biased toward specific temporal intervals or incidental noise patterns, thereby enhancing the operational robustness of the proposed framework across different wind scenarios. The processed dataset was subsequently divided into training, validation, and testing subsets according to chronological order for model development and performance evaluation.

### 3.2. Data Preprocessing and Augmentation Strategy

In multimodal sensor-driven wind power forecasting tasks, raw data are typically collected from heterogeneous meteorological sensors and turbine operational sensors. Such data exhibit characteristics including inconsistent sampling frequencies, strong noise interference, uneven missing value ratios, and significant dimensional disparities. Without systematic data preprocessing and task-oriented data augmentation, deep models may learn incidental noise patterns rather than underlying physical laws, thereby degrading generalization capability. Therefore, prior to constructing the attention-based multimodal sensor importance perception forecasting framework, it is necessary to perform systematic preprocessing from the perspectives of data distribution stability, temporal continuity, and cross-modal consistency, while designing time-series-oriented augmentation strategies to enhance model robustness and transferability under complex wind conditions and diverse operational scenarios. Data preprocessing is first established upon the principle of temporal sequence consistency. Since sampling intervals vary across different sensors, temporal alignment must be conducted to project all modalities onto a unified time axis $\left\{t_{1},t_{2},\ldots ,t_{N}\right\}$. Let the raw observation sequence of the *m*-th sensor modality be denoted as $x^{\left(m\right)}\left(t\right)$, and the aligned sequence be obtained through a temporal resampling operator R(·), yielding ${\tilde{x}}^{\left(m\right)}\left(t_{i}\right)$. Common resampling approaches include linear interpolation and spline interpolation. Linear interpolation can be expressed as:

(1) $$\tilde{x}\left(t\right)=x\left(t_{a}\right)+\frac{t-t_{a}}{t_{b}-t_{a}}\left[x\left(t_{b}\right)-x\left(t_{a}\right)\right],$$

where $t_{a}<t<t_{b}$ denote adjacent valid sampling timestamps. Through temporal alignment, multimodal sensor observations can participate in fusion and attention weight allocation at identical time steps, maintaining consistency with the dynamic sensor importance modeling objective. Following temporal alignment, data cleaning is conducted based on statistical robustness principles. Wind farm sensors are prone to spike noise and distorted readings under extreme wind, icing conditions, or electromagnetic interference. If such outliers are directly incorporated into model training, gradient-based optimization processes may be adversely affected. Outlier detection is commonly performed based on statistical distribution or local density estimation. The 3σ criterion can be formulated as:

(2) $$|x_{i}-\mu |>3\sigma ,$$

where μ and σ denote sample mean and standard deviation, respectively. For non-Gaussian distributions, a more robust method is based on the interquartile range IQR:

(3) $$x_{i}<Q_{1}-1.5\times IQR\;\mathrm{or}\;x_{i}>Q_{3}+1.5\times IQR,$$

where $IQR=Q_{3}-Q_{1}$. Instead of direct deletion, detected outliers are typically replaced using temporal contextual information to preserve sequence continuity. A common smoothing strategy is local window median or weighted mean filtering:

(4) $${\hat{x}}_{t}=\frac{1}{2k+1}\sum_{i=-k}^{k}x_{t+i}.$$

This smoothing process essentially functions as a low-pass filter, preserving wind condition trends while suppressing high-frequency noise. Missing value imputation is grounded in temporal correlation and cross-modal dependency principles. Let the observation sequence be denoted as $x_{t}$, and the missing indicator variable be $m_{t}\in \left\{0,1\right\}$, where $m_{t}=0$ indicates missingness. The simplest interpolation approach estimates missing values using neighboring observations:

(5) $${\hat{x}}_{t}=\frac{x_{t-1}+x_{t+1}}{2}.$$

However, multimodal wind power data exhibit cross-sensor coupling relationships. For instance, nonlinear functional dependencies exist between wind speed and power output. Therefore, regression-based imputation models can be constructed:

(6) $${\hat{x}}_{t}^{\left(m\right)}=f\left(x_{t}^{\left(1\right)},x_{t}^{\left(2\right)},\ldots ,x_{t}^{\left(M\right)}\right),$$

where f(·) can be implemented using lightweight neural networks or ridge regression models. This strategy aligns with the multimodal fusion paradigm by introducing inter-modal dependency structures at the data level, thereby alleviating the learning burden of subsequent attention modules. Normalization is performed based on the principles of numerical stability, gradient scale consistency, and dimensional consistency across the multi-source dataset. As detailed in Table 2, the measurements from the integrated energy system exhibit significant disparities in magnitude, such as wind speed in m/s, air pressure in hPa, and active power in kW. Without proper scaling, direct model input would bias gradient updates toward high-magnitude features and disrupt the learning of physical correlations. To establish dimensional consistency, min-max normalization is employed as follows:

(7) $$x_{i}^{\prime }=\frac{x_{i}-x_{\min}}{x_{\max}-x_{\min}}.$$

Alternatively, standardization is used to transform the heterogeneous data into zero-mean unit-variance distributions:

(8) $$x_{i}^{\prime }=\frac{x_{i}-\mu }{\sigma }.$$

By ensuring dimensional consistency through these techniques, the framework prevents the attention weights in the multimodal fusion module from being dominated by the raw numerical scales of specific modalities. This unification is essential for objective sensor importance learning, as it allows the model to prioritize sensors based on their physical information content rather than their measurement units. After completing fundamental cleaning and scale unification, data augmentation is further introduced to enhance model generalization under complex wind conditions. Time-series augmentation is grounded in distribution perturbation consistency, whereby equivalent perturbed samples are generated without altering physical semantics. To ensure that the generated samples strictly adhere to the physical laws of wind power generation, particularly the power law where output is proportional to the cube of wind speed, we implement a physics-informed data augmentation strategy. Specifically, we incorporate a physical consistency constraint into the noise injection process to ensure that synthetic samples remain within the theoretical power curve. A method of constrained noise injection is adopted, where zero-mean stochastic noise $\epsilon_{t}$ is added to the original observation $x_{t}$:

(9) $${\tilde{x}}_{t}=x_{t}+\epsilon_{t},\;\epsilon_{t}∼N\left(0,\sigma^{2}\right).$$

To prevent the model from learning unphysical patterns such as power dropping while wind speed increases, a monotonicity-preserving filter is applied to the augmented pairs. This strategy simulates sensor measurement errors and improves robustness to real-world noise environments while ensuring that the relationship between wind speed and power output remains consistent with the underlying atmospheric physics. To model temporal dependency structures, sliding window slicing and recombination augmentation can also be applied. Let the original sequence be a length-*T* temporal window $X_{1:T}$. Sub-sequences are constructed using a sliding operator W:

(10) $$X^{\left(i\right)}=\left\{x_{i},x_{i+1},\ldots ,x_{i+L-1}\right\}.$$

By varying window starting index *i* and length *L*, multi-scale training samples can be generated, enabling the model to simultaneously learn short-term fluctuations and mid-term trend dynamics, which is particularly important under rapidly changing wind conditions. Considering collaborative relationships among multimodal sensors, modality-consistent augmentation can also be introduced through joint perturbation. For a multimodal vector $x_{t}$ at time *t*, proportional scaling is applied:

(11) $${\tilde{x}}_{t}=\alpha x_{t},\;\alpha \in \left[1-\delta ,1+\delta \right].$$

This approach simulates global environmental intensity variations, such as overall wind field strengthening or weakening, facilitating stable learning of relative importance distributions within attention modules. In addition, temporal warping augmentation can be employed to simulate variability in wind speed evolution rates. Let the temporal mapping function be τ(t), generating a nonlinear time axis:

(12) $$\tilde{x}\left(t\right)=x\left(\tau \left(t\right)\right),$$

where τ(t) is a monotonic function. This strategy produces wind condition samples with diverse temporal dynamics, enhancing adaptability to non-stationary temporal structures. Through the aforementioned multi-level preprocessing and augmentation strategies, the dataset is optimized in terms of statistical distribution, temporal continuity, and cross-modal consistency before being input into the multimodal attention fusion model. This process not only mitigates misleading effects caused by noise and missing values but also expands the effective sample space via structured augmentation. Consequently, the subsequent sensor importance perception attention module is enabled to learn more stable and physically consistent weight distribution patterns, thereby establishing a reliable data foundation for high-reliability wind power forecasting in green energy scenarios.

### 3.3. Proposed Method

#### 3.3.1. Overall

The proposed framework employs a hierarchical modeling strategy to transform raw, heterogeneous multimodal sensor streams into a high-precision power forecast. For a given set of *M* modality sequences ${\left\{X_{t-L+1:t}^{\left(m\right)}\right\}}_{m=1}^{M}$ defined within a look-back window *L*, the model generates an *H*-step trajectory ${\hat{y}}_{t+1:t+H}$. As delineated in Algorithm 1, the architecture is centered on three core modeling contributions: intra-modal dynamic representation, cross-modal importance perception, and multi-horizon regression. Initially, parallel encoders project each modality into a shared latent space to extract temporal patterns $H^{\left(m\right)}$, a step that effectively isolates modality-specific dynamics while preserving physical heterogeneity.

Subsequently, the sensor-importance-aware module functions as the central fusion hub, adaptively learning modality weights α through relational embeddings. This process allows the framework to dynamically assess the reliability of different sensing sources under varying wind conditions. The resulting fused representation Z is formulated as a convex combination of modality-specific features, which serves to suppress irrelevant noise and amplify critical predictors. The forecasting head then aggregates these representations to produce the multi-step output $\hat{y}$. By jointly optimizing these components, the framework achieves a balance between predictive performance across multiple temporal horizons and the end-to-end interpretability provided by the attribution weights α.
**Algorithm 1** Overall Workflow of the Multimodal Sensor-Importance-Aware Forecasting**Require:** 
Multimodal input sequences $\left\{X^{\left(m\right)}\right\}$, Window length *L*, Forecasting horizon *H***Ensure:** 
Power forecast $\hat{y}$, Sensor importance weights α1:**for** each modality m∈{1,…,M} **do**2:   Project $X^{\left(m\right)}$ into shared latent space to obtain embeddings $E^{\left(m\right)}$;3:   Extract temporal patterns $H^{\left(m\right)}$ using one-dimensional convolutional operators;4:   Compute modality summary $s^{\left(m\right)}$ via temporal aggregation;5:**end for**6:Construct adjacency matrix A and learn relational embeddings $e_{m}$;7:Calculate importance weights α through modality-wise normalization;8:Form fused representation Z as a convex combination of $\left\{H^{\left(m\right)}\right\}$;9:Map integrated representation Y to the multi-step forecast $\hat{y}$;10:Return $\hat{y}$ and joint attribution map Γ;

#### 3.3.2. Multi-Modal Sensor Feature Encoding Module

The preprocessed multimodal time-window samples are organized as a tensor $X\in R^{M\times L\times F}$, where *F* denotes the collection of raw feature dimensions.

**Figure 1:**
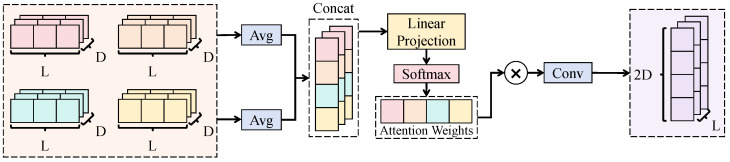
Schematic illustration of the multi-modal sensor feature encoding module.

As shown in Figure 1, to eliminate scale discrepancies and facilitate cross-modal alignment, the *m*-th modality input $X^{\left(m\right)}\in R^{L\times F_{m}}$ is first mapped into a shared latent space to obtain $E^{\left(m\right)}\in R^{L\times d}$ through an intra-modal embedding composed of multi-layer feed-forward transformations:

(13) $$E^{\left(m\right)}=f_{\mathrm{emb}}^{\left(m\right)}\;\left(X^{\left(m\right)}\right).$$

Then, to extract intra-modal local dynamics and temporal dependencies, a temporal encoder constructed by multiple one-dimensional convolutional operators is employed to map $E^{\left(m\right)}$ into $H^{\left(m\right)}\in R^{L\times d}$:

(14) $$U_{r}^{\left(m\right)}=\sigma \;\left(\mathrm{Conv}1D_{k_{r},c_{r}}\;\left(U_{r-1}^{\left(m\right)}\right)\right),\;U_{0}^{\left(m\right)}=E^{\left(m\right)},\;H^{\left(m\right)}=\mathrm{LN}\;\left(U_{R}^{\left(m\right)}\right),$$

where *R* denotes the encoding depth, $\left\{k_{r}\right\}$ and $\left\{c_{r}\right\}$ are hyperparameters for kernel scales and channel widths, respectively, σ(·) is an element-wise nonlinearity, and LN(·) denotes layer normalization. To interface with modality-level importance modeling in the subsequent fusion stage, a modality summary vector $s^{\left(m\right)}\in R^{d}$ is further constructed to characterize the overall state of the modality within the time window:

(15) $$s^{\left(m\right)}=A\;\left(H^{\left(m\right)}\right),$$

where A(·) denotes a temporal aggregation operator. Finally, stacked modality features are represented as $H\in R^{M\times L\times d}$, which serves as the input to the sensor-importance-aware attention fusion module. In this way, an information flow of intra-modal representation learning → modality-level summarization → cross-modal importance allocation is established, providing a foundation for interpretability and robustness in the subsequent fusion stage.

#### 3.3.3. Sensor-Importance-Aware Attention Fusion Module

The sensor-importance-aware attention fusion module receives the stacked modality features $H\in R^{M\times L\times d}$ and modality summaries $\left\{s^{\left(m\right)}\right\}$, and learns an importance allocation $\alpha \in R^{M}$ by treating sensor modalities as the attention objects. Different from self-attention mechanisms that model dependencies among sequence tokens via *Q*–*K* similarity, the normalization in this module is performed along the modality dimension, thereby directly producing interpretable sensor contribution proportions and avoiding the attribution drift that can occur with token-level attention in long sequences.

**Figure 2:**
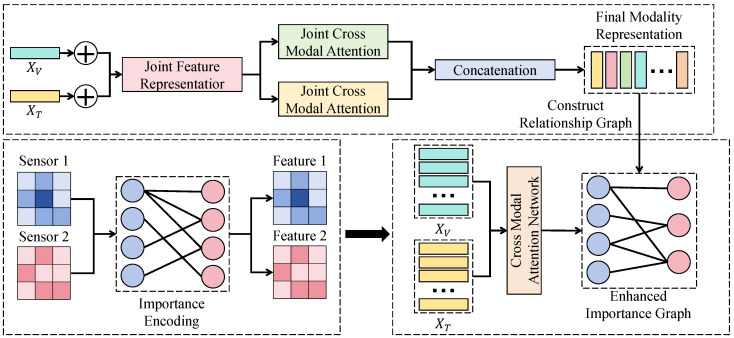
Schematic illustration of the sensor-importance-aware attention fusion module.

As shown in Figure 2, modality summaries are first mapped into relational-space embeddings $e_{m}$:

(16) $$e_{m}=f_{\mathrm{imp}}\;\left(s^{\left(m\right)}\right).$$

Then, a weighted adjacency matrix $A\in R^{M\times M}$ is constructed across modality nodes to characterize inter-sensor coordination:

(17) $$s_{mn}=\frac{e_{m}^{⊤}Ue_{n}}{\sqrt{d_{r}}},\;A_{mn}=\frac{\exp(s_{mn})}{\sum_{j=1}^{M}\exp\left(s_{mj}\right)},$$

where $d_{r}$ denotes the relational embedding dimension and U is a learnable parameter. Based on A, a relation-enhanced representation ${\tilde{s}}^{\left(m\right)}$ is obtained and projected to an importance logit, followed by modality-wise normalization to yield α:

(18) $${\tilde{s}}^{\left(m\right)}=\sum_{n=1}^{M}A_{mn}s^{\left(n\right)},\;u_{m}=g\;\left({\tilde{s}}^{\left(m\right)}\right),\;\alpha_{m}=\frac{\exp(u_{m})}{\sum_{j=1}^{M}\exp\left(u_{j}\right)}.$$

This cross-modal relational modeling essentially functions as an endogenous sensor integrity check. Although a drifting sensor might remain correlated with the power output, its physical consistency relative to other heterogeneous modalities will inevitably decrease. The weighted adjacency matrix A captures such relational anomalies by measuring the coordination between modality nodes. If a primary sensor exhibits calibration drift, its relational embedding $e_{m}$ will deviate from the learned physical manifold, leading to a reduction in its relative importance weight $\alpha_{m}$ within the global context. This mechanism ensures that the framework can adaptively suppress systematic errors by leveraging the collective reference of the entire sensor suite. Finally, α is used to re-calibrate modality features and to aggregate them along the modality dimension to obtain the fused temporal representation $Z\in R^{L\times d}$:

(19) $$Z_{t,:}=\sum_{m=1}^{M}\alpha_{m}\;H_{m,t,:}.$$

Since $\alpha_{m}\geq 0$ and $\sum_{m=1}^{M}\alpha_{m}=1$, $Z_{t,:}$ is a convex combination of modality features at each time step, which suppresses the destructive impact of anomalous fluctuations from noisy modalities and provides consistent and comparable sensor-importance outputs for subsequent forecasting and interpretability analysis.

#### 3.3.4. Wind Power Forecasting and Interpretability Analysis Module

The forecasting and interpretability analysis module takes the fused temporal representation $Z\in R^{L\times d}$ as input and forms the final forecasting input together with a compensation.

**Figure 3:**
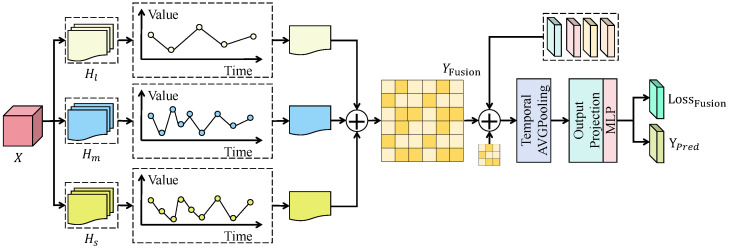
Schematic illustration of the wind power forecasting and interpretability analysis module.

As shown in Figure 3, let the output of the compensation branch be $Z_{h}\in R^{L\times d}$, and the integrated input $Y\in R^{L\times d}$ is defined as

(20) $$Y=Z+Z_{h}.$$

A temporal aggregation operator T(·) is then applied to obtain a global semantic vector $q\in R^{d}$, followed by output projection and regression mapping to generate the multi-step forecast $\hat{y}\in R^{H}$:

(21) $$q=T\left(Y\right),\;h=f_{\mathrm{pred}}\left(q\right),\;\hat{y}=g_{\mathrm{out}}\left(h\right).$$

To enhance reliability under highly volatile wind conditions, scale parameters can be additionally produced and probabilistic objectives can be adopted during training. Let the outputs be the location parameter $\hat{\mu }$ and the scale parameter ${\hat{\sigma }}^{2}$, and the forecasting loss can be formulated as the negative log-likelihood:

(22) $$L_{\mathrm{pred}}=\frac{1}{2}\sum_{i=1}^{H}\left(\frac{{\left(y_{i}-{\hat{\mu }}_{i}\right)}^{2}}{{\hat{\sigma }}_{i}^{2}}+\log{\hat{\sigma }}_{i}^{2}\right).$$

When ${\hat{\sigma }}_{i}^{2}>0$ is fixed, strict convexity with respect to ${\hat{\mu }}_{i}$ is obtained, and the second derivative is given by

(23) $$\frac{\partial^{2}L_{\mathrm{pred}}}{\partial {\hat{\mu }}_{i}^{2}}=\frac{1}{{\hat{\sigma }}_{i}^{2}}>0,$$

which guarantees a unique optimum for mean prediction under a given uncertainty characterization, while ${\hat{\sigma }}_{i}^{2}$ enables adaptive adjustment over high-uncertainty intervals to improve overall stability. For interpretability, the sensor importance α output from the previous module is jointly used with time-contribution weights $\beta \in R^{L}$ read from Y, where β is produced by a temporal readout network and normalized along the time dimension:

(24) $$\beta_{t}=\frac{\exp(\eta_{t})}{\sum_{j=1}^{L}\exp\left(\eta_{j}\right)},\;\eta_{t}=r_{\mathrm{time}}\left(Y_{t,:}\right).$$

The joint attribution coefficient is defined as $\Gamma_{m,t}=\alpha_{m}\beta_{t}$ and satisfies attribution conservation:

(25) $$\sum_{m=1}^{M}\sum_{t=1}^{L}\Gamma_{m,t}=\left(\sum_{m=1}^{M}\alpha_{m}\right)\left(\sum_{t=1}^{L}\beta_{t}\right)=1.$$

Therefore, $\Gamma_{m,t}$ can be directly interpreted as the relative contribution of sensor *m* at time step *t* to the forecast, enabling the construction of a sensor–time two-dimensional contribution map and, together with uncertainty outputs, supporting operational diagnosis and dispatch risk assessment, thereby unifying forecasting accuracy, reliability, and interpretability in green-energy scenarios.

## 4. Results and Discussion

### 4.1. Experimental Configuration

#### 4.1.1. Hardware and Software Platform

The experiments were conducted on a heterogeneous computing platform featuring an Intel Xeon Gold 6448Y processor and an NVIDIA RTX 4090 GPU with 24 GB memory. This system is equipped with 128 GB RAM and a 2 TB SSD to ensure high-speed multi-source data throughput. The software environment is built on Ubuntu 22.04.3 LTS. We utilize Python 3.11.5 and the PyTorch 2.1.0 framework with CUDA 12.1 and cuDNN 8.9.2 acceleration. Supporting library versions include NumPy 1.24.4, Pandas 2.0.3, Scikit-learn 1.3.0, and Matplotlib 3.7.2.

Regarding hyperparameter configuration, the dataset is chronologically split in a 70/15/15 ratio. Training employs a sliding window L = 96 and horizons H = 1 to 12. The Adam optimizer is used with an initial learning rate of 0.001 and a decay factor of 0.5. Batch size is set to 64, maximum epochs to 100 with early stopping, and the attention hidden dimension to d = 128. A 5-fold cross-validation strategy is adopted to ensure robust evaluation across diverse wind scenarios and sample distributions.

#### 4.1.2. Baseline Models and Evaluation Metrics

Representative baseline models from multiple technical paradigms were selected in the experiments to comprehensively validate the effectiveness and advancement of the proposed method through cross-framework comparisons. The Persistence model is constructed upon the short-term inertia characteristics of time series and achieves rapid forecasting by directly propagating the most recent observation, exhibiting advantages in implementation simplicity and response efficiency. The ARIMA model, integrating autoregressive and moving-average structures, is capable of characterizing linear temporal dependency relationships and demonstrates robust performance in stationary sequence modeling. Support vector regression (SVR) relies on kernel mapping mechanisms to project data into high-dimensional feature spaces, where nonlinear regression relationships can be effectively learned with strong generalization capability. Random forest (RF) constructs segmented fitting structures through ensemble decision trees, enabling stable modeling of complex nonlinear features, whereas gradient boosting decision tree progressively optimizes residual learning trajectories via gradient boosting strategies, achieving superior regression accuracy. Long short-term memory establishes long- and short-term memory pathways through gated recurrent units, allowing historical wind condition information to be continuously accumulated to enhance temporal modeling capacity. Gated recurrent unit (GRU) maintains memory mechanisms while adopting a more compact structure, thereby achieving a favorable balance between computational efficiency and predictive performance. Temporal convolutional network (TCN) constructs large receptive-field temporal modeling frameworks based on dilated convolutions, combining parallel computation advantages with strong long-range dependency capture capability. Transformer directly models global temporal dependency relationships through self-attention mechanisms, enabling adaptive focus on critical time-step features and demonstrating outstanding performance in complex wind condition forecasting tasks. Evaluation metrics were employed to quantify forecasting error and fitting capability from multiple perspectives. Mean absolute error was used to measure overall deviation magnitude, root mean square error characterized sensitivity to large deviations, mean absolute percentage error reflected relative error levels, and the coefficient of determination evaluated global fitting quality and explanatory capacity. The mathematical definitions of the commonly used metrics are given as follows:

(26) $$MAE=\frac{1}{N}\sum_{i=1}^{N}\left|y_{i}-{\hat{y}}_{i}\right|,$$

(27) $$RMSE=\sqrt{\frac{1}{N}\sum_{i=1}^{N}{\left(y_{i}-{\hat{y}}_{i}\right)}^{2}},$$

(28) $$MAPE=\frac{100\%}{N}\sum_{i=1}^{N}\left|\frac{y_{i}-{\hat{y}}_{i}}{y_{i}+\epsilon }\right|,$$

(29) $$R^{2}=1-\frac{\sum_{i=1}^{N}{\left(y_{i}-{\hat{y}}_{i}\right)}^{2}}{\sum_{i=1}^{N}{\left(y_{i}-\bar{y}\right)}^{2}},$$

where *N* denotes the number of test samples, $y_{i}$ represents the ground-truth wind power at the *i*-th time step, ${\hat{y}}_{i}$ denotes the predicted value, $\bar{y}$ is the mean of the ground-truth sequence, and ϵ is a small constant introduced to avoid division-by-zero instability when power values approach zero.

### 4.2. Overall Performance Comparison with Baseline Models

The objective of this experiment is to systematically evaluate the overall performance advantages of the proposed multimodal sensor-importance-aware forecasting framework under a unified data setting and evaluation metric system. Through horizontal comparisons with representative baseline models of different methodological paradigms, the effectiveness of the proposed architecture in nonlinear representation, temporal dependency modeling, and multi-source information fusion is comprehensively validated. The reported results span the full methodological spectrum from conventional statistical approaches and classical machine learning models to advanced deep learning architectures, thereby providing a holistic reflection of model adaptability in wind power forecasting scenarios characterized by strong randomness and volatility.

**Figure 4:**
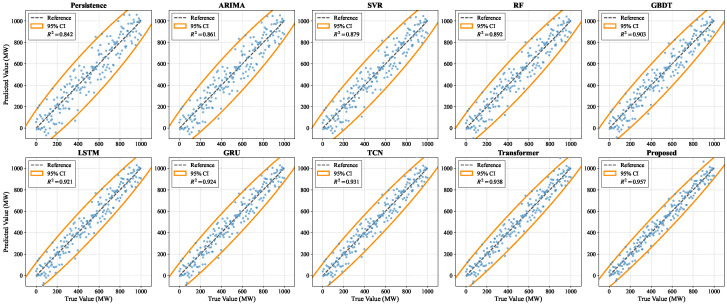
Scatter distribution and fitting consistency comparison between true and predicted wind power values (MW) for nine baseline models and the proposed method. The horizontal axis represents the true wind power values while the vertical axis denotes the predicted values, with a dashed reference line representing the ideal forecasting scenario. Each subplot further incorporates orange shaded regions to illustrate the 95% confidence intervals and displays the $R^{2}$ value to quantify the overall fitting performance. The notably tighter concentration of data points along the diagonal in the Proposed Method subplot, compared to standard deep learning and statistical benchmarks, demonstrates its enhanced capability in modeling non-stationary wind power fluctuations.

As shown in Table 3 and Figure 4, the Persistence method exhibits the weakest performance across all evaluation metrics, as forecasting is performed solely through extrapolation from the most recent observation without modeling wind speed variability or turbine response lag effects. ARIMA introduces linear temporal dependency modeling through differencing and autoregressive structures; however, its reliance on stationarity assumptions and linear superposition limits its capability to capture complex nonlinear fluctuations driven by meteorological coupling. SVR improves performance by projecting data into high-dimensional feature spaces through kernel mappings, enabling nonlinear regression learning, yet its static formulation restricts effective modeling of long-range temporal dependencies. Tree-based ensemble models such as RF and GBDT demonstrate stronger nonlinear fitting capability through piecewise partitioning of the feature space. GBDT further improves performance via iterative residual optimization, though both approaches lack explicit temporal memory mechanisms and remain limited in modeling cross-scale temporal dynamics. With the introduction of deep learning models, forecasting performance improves significantly. LSTM and GRU establish long- and short-term memory propagation paths through gated recurrent structures, enabling historical wind condition accumulation within hidden states. GRU achieves slightly better generalization under limited sample conditions due to its more compact parameterization. TCN expands the temporal receptive field via dilated convolutions, enhancing long-range dependency capture while maintaining parallel computation efficiency. Transformer further advances performance by directly modeling global temporal dependencies through self-attention, adaptively focusing on critical time intervals under complex wind conditions. In contrast, the proposed method first performs intra-modal dynamic representation learning and subsequently allocates information contributions across modalities through sensor-importance-aware attention, while cross-modal relational modeling suppresses redundant noisy modalities. The fusion representation therefore achieves improved signal-to-noise characteristics and stronger physical consistency. Combined with prediction-side compensation and uncertainty characterization mechanisms, the proposed framework attains the best performance across all metrics. From a mathematical perspective, this improvement stems from the decoupled yet collaborative optimization of temporal dependency modeling and modality contribution modeling, structurally enhancing both discriminative representation capacity and interpretability within the feature space, thereby yielding superior fitting capability and generalization stability for complex non-stationary wind power sequences.

### 4.3. Performance Comparison Under Different Forecasting Horizons

The purpose of this experiment is to systematically evaluate model adaptability across multiple forecasting horizons and to analyze error accumulation behavior under multi-step prediction settings. By progressively extending the prediction horizon and comparing performance degradation trends, model stability and generalization capability under short-term and medium-to-long-term forecasting scenarios are assessed. Given the strong non-stationarity and stochastic volatility inherent in wind power sequences, longer prediction horizons typically amplify uncertainty and propagate errors; therefore, single-horizon evaluations are insufficient to characterize practical forecasting value. Multi-horizon settings enable observation of performance decay patterns under varying temporal dependency intensities.

**Figure 5:**
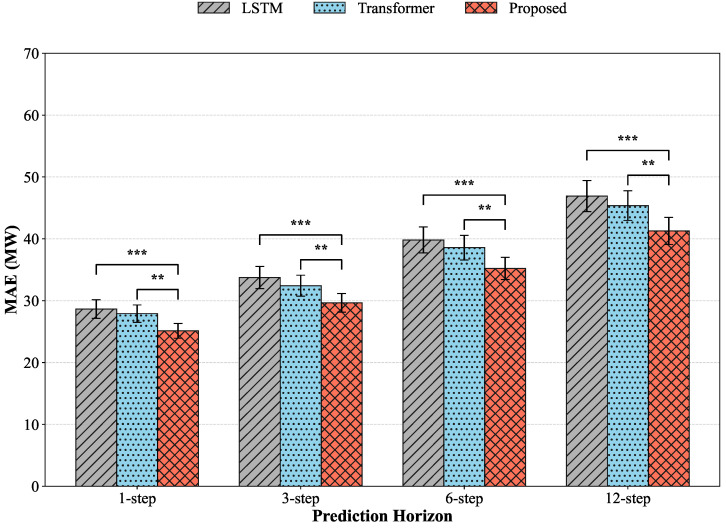
Boxplot comparison of RMSE distributions and statistical significance across different model variants in the wind power forecasting task. Significance levels are indicated by * (*p* < 0.05), ** (*p* < 0.01), and *** (*p* < 0.001) compared to the baseline.

As shown in Table 4 and Figure 5, all models achieve relatively strong performance under single-step forecasting conditions, with Transformer outperforming LSTM due to its global dependency modeling capability. The proposed method further reduces prediction errors and improves goodness-of-fit, indicating that short-term forecasting benefits not only from temporal information but also from complementary inter-sensor features introduced through sensor-importance-aware modeling. As the forecasting horizon extends, performance degradation is observed across all models; however, the error growth rate of the proposed framework remains significantly lower than that of baseline architectures, demonstrating stronger robustness against error propagation. From a theoretical perspective, these differences arise from structural variations in temporal dependency modeling pathways and information utilization strategies. Recurrent architectures rely on hidden-state propagation to encode historical dynamics, making them effective for short-term response modeling but increasingly susceptible to accumulated noise and bias as prediction steps expand. Self-attention architectures capture long-range dependencies more effectively through direct global interactions, yielding improved medium-term performance, yet remain constrained to temporal token relationships without explicit modeling of cross-sensor physical contribution heterogeneity. The proposed framework incorporates sensor-importance perception and cross-modal relational modeling prior to prediction, suppressing redundant noise sources at the feature fusion stage and reducing upstream error propagation. Prediction-side compensation and uncertainty characterization further enhance tolerance to abrupt wind condition fluctuations, enabling more stable forecasting performance under extended horizons. Consequently, simultaneous optimization of temporal representation and modality contribution allocation results in slower performance decay and improved reliability across multi-horizon forecasting tasks.

### 4.4. Ablation Study

The ablation experiment is designed to quantitatively evaluate the contribution of each core architectural component within the proposed framework. By progressively removing or modifying key modules under identical training and testing settings, performance variations are observed to reveal the functional role of each structural design in error suppression and predictive capability enhancement.

**Figure 6:**
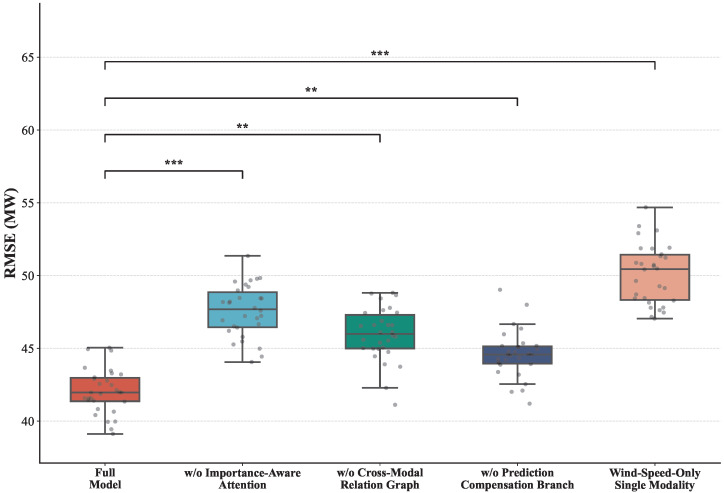
Boxplot comparison of RMSE distributions and statistical significance across different model variants in the wind power forecasting task. Significance levels are indicated by * (*p* < 0.05), ** (*p* < 0.01), and *** (*p* < 0.001) compared to the baseline.

As shown in Table 5 and Figure 6, the complete framework achieves the best performance across all metrics, confirming the synergistic gain formed by multimodal encoding, importance-aware fusion, relational modeling, and prediction compensation. The most significant degradation occurs when the sensor-importance-aware attention mechanism is removed, indicating that without dynamic contribution allocation, multimodal features are fused in a static or averaged manner, allowing redundant noise to propagate into the prediction stage. Removing the cross-modal relation graph also degrades performance, though to a lesser extent, suggesting that relational modeling serves as an enhancement layer refining importance allocation rather than a foundational component. From the prediction-structure perspective, removing the compensation branch results in moderate performance decline, reflecting its stabilizing role in handling short-term abrupt fluctuations and turbine inertia lag effects. The single-modality setting yields the largest degradation, demonstrating that wind power generation is driven by complex nonlinear interactions between meteorological conditions and turbine operational states rather than any single variable. Structurally, the full framework performs intra-modal temporal compression, inter-modal importance weighting, relational reconstruction, and prediction-side compensation in a progressive denoising and enhancement pipeline. This hierarchical information refinement improves signal-to-noise characteristics and stabilizes gradient optimization, constituting the fundamental reason for its superior performance over all ablation variants.

### 4.5. Discussion

In practical wind power operation scenarios, power forecasting is not merely a matter of model accuracy but is directly associated with grid dispatching security and renewable energy accommodation efficiency. Taking large-scale grid-connected wind power bases as an example, dispatching centers are required to formulate unit output scheduling plans and reserve capacity allocation strategies based on forecasting results over the next several hours or even longer temporal horizons. When forecasting deviations become excessive, temporary compensation is often required through thermal power generation or energy storage systems, which not only increases dispatching costs but also weakens the substitution benefits of clean energy.

To provide practical guidance for selecting appropriate forecasting tools, we offer specific recommendations for different model application cases. For ultra-short-term scenarios characterized by high stationarity and minimal computational resources, statistical baselines like Persistence or ARIMA are recommended due to their rapid response and simplicity. In scenarios where historical data is limited but nonlinear mapping is required, machine learning models such as GBDT or SVR remain competitive for moderate-scale fitting tasks. For large-scale wind farms with high-frequency data streams, deep learning architectures like LSTM or Transformer are suitable for capturing long-range temporal dependencies. However, our proposed multimodal sensor-importance-aware framework is specifically recommended for complex operational environments subject to strong convective weather or abrupt seasonal transitions. In such cases, the dynamic weight allocation mechanism effectively suppresses noise from unreliable sensors while highlighting critical driving factors, providing the high-level robustness and interpretability required for safety-sensitive grid dispatching and real-time frequency regulation.

Regarding the engineering feasibility, we emphasize that the proposed framework is designed with a focus on both high accuracy and deployment efficiency. While high-performance hardware was utilized during the offline training and multi-fold cross-validation phases to accelerate experimental iterations, the actual inference process is computationally efficient. The core components, including the one-dimensional convolutional encoders and the linear projection-based relational graph, involve lightweight matrix operations that do not require massive memory or high-end GPU resources for single-sample prediction. As demonstrated in our updated performance analysis in Table 2, the inference time for a single prediction remains within a millisecond range, which is well below the 10-min sampling interval of typical wind farm SCADA systems. Furthermore, the model architecture is compatible with mainstream model compression techniques such as quantization and knowledge distillation, facilitating its integration into lightweight industrial controllers or edge computing nodes located directly at the turbine. This ensures that the framework can satisfy the real-time processing requirements of localized energy management while maintaining a low computational footprint.

From the perspective of wind farm operation and maintenance management, the proposed framework also demonstrates direct application value. The learned importance weights exhibit a high degree of alignment with physical insights derived from wind turbine control logic and aerodynamic principles. Specifically, we observe that the dynamic distribution of weights correlates closely with different stages of the power curve. In the region below the rated wind speed, the model naturally assigns higher weights to turbine internal state variables such as rotor speed and active power, which is consistent with the Maximum Power Point Tracking (MPPT) control objectives where mechanical dynamics are the primary drivers of output. In contrast, during periods of high wind speed or severe weather transitions, the importance weights shift toward external meteorological variables such as air pressure gradients and ultrasonic wind speed measurements. This transition matches the physical reality where pitch control mechanisms and atmospheric stability classes become the dominant factors limiting or determining power generation. Through long-term statistical analysis of sensor importance weights, the configuration rationality of wind measurement systems and operational monitoring infrastructures can be reversely evaluated, providing quantitative guidance for sensor deployment optimization and equipment maintenance. Moreover, in curtailment management or power smoothing control scenarios, dispatchers are often required to determine whether power fluctuations are primarily driven by external wind variations or internal turbine state constraints. The cross-modal contribution distributions generated by the proposed framework offer intuitive support for such decision-making processes. Furthermore, within power systems characterized by high renewable penetration, wind power forecasting outcomes directly influence energy storage charging–discharging strategies and electricity market spot trading quotations. Improvements in forecasting accuracy and reliability contribute to reduced reserve capacity requirements and lower trading risks, thereby achieving a more favorable balance between economic efficiency and operational security. Consequently, across multiple practical business segments, including grid-connected wind power dispatching, equipment operation diagnostics, and renewable energy market operations, the proposed method demonstrates clear engineering deployment significance and support value for green energy systems.

### 4.6. Limitation and Future Work

Although a relatively comprehensive wind power forecasting framework centered on multimodal sensor importance perception modeling has been established, and its effectiveness has been validated through multi-horizon and multi-baseline comparative experiments, further extension and refinement remain necessary. First, the current model relies heavily on the data quality and spatial coverage density of deployed sensor infrastructures. When wind measurement towers are sparsely distributed or when certain turbine sensors experience long-term drift, the reliability of importance allocation results may be constrained by input data fidelity. Second, the issue of temporal latency and asynchronicity between heterogeneous data sources requires further investigation. In this study, we utilize aligned historical datasets to validate the architectural potential of the importance-aware mechanism. However, in real-world wind farm operations, Numerical Weather Prediction (NWP) data often arrives with a significant delay of 1 to 6 h, while SCADA data is collected in real time. Our current assumption of unified timestamps through resampling might not fully reflect live deployment performance, where the most critical meteorological features could be stale. Future research will focus on developing latency-robust fusion strategies and asynchronous modeling techniques to bridge the gap between retrospective validation and real-time industrial requirements. Third, the proposed framework primarily focuses on centralized modeling at the single wind farm scale. The characterization of inter-farm spatial correlations, wake effects, and regional meteorological co-evolution processes remains insufficient, as explicit spatial topological structures and terrain constraint information have not yet been incorporated. In addition, although attention weights provide a degree of interpretability, the causal consistency between learned importance distributions and underlying physical mechanisms still requires further cross-validation through field experiments and physics-based models. Future research may explore directions such as multi-wind-farm collaborative forecasting, integration of numerical weather prediction data, and lightweight deployment on edge-side devices. By constructing cross-scale spatiotemporal coupling modeling mechanisms and energy-aware inference strategies, forecasting performance, computational efficiency, and engineering deployability could be jointly optimized under ultra-large-scale renewable grid integration environments.

## 5. Conclusions

Under the accelerated transition of green energy structures, forecasting accuracy has become a critical technical foundation for the security and efficiency of nonlinear multi-source integrated energy systems. This study constructed a wind power forecasting framework based on multimodal sensor importance perception to address challenges in quantifying differential information contributions and modeling strong data heterogeneity. At the architectural level, the collaborative optimization of temporal dependency and modality allocation allows meteorological and turbine data to be transformed into unified representations while preserving physical differences. A key finding of this research is that the learned dynamic weights are closely aligned with the physical operating states of the energy system. Specifically, the model identifies the transition of dominance from mechanical dynamics during maximum power point tracking stages to meteorological gradients during high-wind transitions, providing deep insights into the nonlinear coupling of the system. Comprehensive experimental results validate the effectiveness of the proposed method. In baseline comparisons, the model achieved an $R^{2}$ of 0.957, markedly superior to the Transformer baseline. Multi-horizon experiments demonstrated that the framework maintains stronger long-term stability than LSTM and Transformer models, particularly in suppressing performance degradation over twelve-step horizons. Ablation studies confirmed that the importance-aware attention component is the core driver of performance enhancement. By unifying forecasting accuracy with physical interpretability, this framework provides a reliable technical basis for the optimized operation of green energy systems and grid-side dispatching, offering actionable insights for the management of complex, multi-source energy infrastructures.

## Figures and Tables

**Table 1:** Comparison of different interpretability and fusion paradigms in wind power forecasting.

Methodology	Interpretability Type	Explanation Target	Fusion Strategy	Modality Awareness
Post-hoc (SHAP/LIME)	External/Post-hoc	Individual Features	Agnostic	None
Informer/Autoformer	Self-interpretable	Temporal Steps	Early Fusion	Token-level only
Gated Lag Selection	Self-interpretable	Lagged Variables	Feature-level	Variable-specific
Proposed Method	Self-interpretable	Sensor Modalities	Relational Fusion	Dynamic Modality-level

**Table 2:** Statistics of multimodal sensor data in the wind farm.

Feature Name	Sensor/Device Source	Valid Samples
wind speed	ultrasonic anemometer	104,386
wind direction	wind vane	104,102
ambient temperature	temperature sensor	103,875
relative humidity	humidity sensor	103,642
air pressure	high-precision barometer	103,214
active power output	electric energy meter	105,120
rotor speed	rotational encoder	104,557
generator speed	rotational speed sensor	104,221
gearbox temperature	thermocouple sensor	103,908
yaw angle	yaw position sensor	103,476

**Table 3:** Overall performance comparison of different baseline models on the wind power forecasting task. Values are presented as mean ± standard deviation from 5-fold cross-validation. Significance levels are indicated by * (*p* < 0.05), ** (*p* < 0.01), and *** (*p* < 0.001) compared to the Transformer baseline. The symbol ↑ signifies that larger values correspond to better model performance, and vice versa.

Method	MAE ↓	RMSE ↓	MAPE (%) ↓	$R^{2}↑$	Inference Time (ms) ↓
Persistence	52.31 ± 1.15	71.84 ± 1.56	18.26 ± 0.42	0.842 ± 0.012	0.01 ± 0.00
ARIMA	47.95 ± 0.98	66.27 ± 1.34	16.03 ± 0.38	0.861 ± 0.010	3.42 ± 0.12
SVR	44.18 ± 0.85	61.74 ± 1.12	14.92 ± 0.35	0.879 ± 0.008	15.18 ± 0.45
RF	41.63 ± 0.76	58.20 ± 1.05	13.57 ± 0.28	0.892 ± 0.007	21.65 ± 0.62
GBDT	39.74 ± 0.68	55.11 ± 0.92	12.98 ± 0.24	0.903 ± 0.006	25.84 ± 0.78
LSTM	36.28 ± 0.62	50.67 ± 0.84	11.42 ± 0.21	0.921 ± 0.005	52.37 ± 1.34
GRU	35.91 ± 0.58	49.88 ± 0.79	11.15 ± 0.19	0.924 ± 0.005	48.12 ± 1.22
TCN	34.75 ± 0.52	48.06 ± 0.71	10.87 ± 0.16	0.931 ± 0.004	38.96 ± 0.95
Transformer	33.62 ± 0.48	46.91 ± 0.65	10.24 ± 0.14	0.938 ± 0.004	74.23 ± 1.86
Proposed Method	30.48 ± 0.35 ***	42.37 ± 0.52 ***	9.16 ± 0.11 **	0.957 ± 0.003 ***	105.58 ± 2.45

**Table 4:** Multi-step wind power forecasting performance under different prediction horizons.

Horizon	Method	MAE ↓	RMSE ↓	MAPE (%) ↓	$R^{2}↑$
1-step	LSTM	28.64	39.85	8.72	0.962
	Transformer	27.91	38.77	8.35	0.966
	Proposed Method	**25.13**	**35.42**	**7.41**	**0.974**
3-step	LSTM	33.75	46.18	10.94	0.938
	Transformer	32.42	44.63	10.31	0.943
	Proposed Method	**29.66**	**41.05**	**9.12**	**0.955**
6-step	LSTM	39.82	54.73	13.87	0.912
	Transformer	38.57	52.94	13.02	0.918
	Proposed Method	**35.21**	**48.37**	**11.48**	**0.934**
12-step	LSTM	46.91	63.84	16.75	0.881
	Transformer	45.38	61.92	15.96	0.889
	Proposed Method	**41.27**	**56.48**	**14.02**	**0.907**

**Table 5:** Ablation study results of key modules.

Model Variant	MAE ↓	RMSE ↓	MAPE (%) ↓	$R^{2}↑$
Full Model	**30.48**	**42.37**	**9.16**	**0.957**
w/o Importance-Aware Attention	33.92	47.81	10.87	0.936
w/o Cross-Modal Relation Graph	32.75	45.94	10.21	0.942
w/o Prediction Compensation Branch	31.86	44.63	9.74	0.949
Wind-Speed Only (Single Modality)	36.41	50.28	11.92	0.921

## Data Availability

The data presented in this study are available on request from the corresponding author.
